# The role of procalcitonin in identifying high‐risk cancer patients with febrile neutropenia: A useful alternative to the multinational association for supportive care in cancer score

**DOI:** 10.1002/cam4.4355

**Published:** 2021-11-01

**Authors:** Patrick Chaftari, Anne‐Marie Chaftari, Ray Hachem, Sai‐Ching J. Yeung, Hiba Dagher, Ying Jiang, Alexandre E. Malek, Natalie Dailey Garnes, Victor E. Mulanovich, Issam Raad

**Affiliations:** ^1^ Department of Emergency Medicine The University of Texas MD Anderson Cancer Center Houston Texas USA; ^2^ Department of Infectious Diseases, Infection Control and Employee Health The University of Texas MD Anderson Cancer Center Houston Texas USA

**Keywords:** cancer patients, febrile neutropenia, immunocompromised, lactate, neutropenia, procalcitonin

## Abstract

**Background:**

The Multinational Association for Supportive Care in Cancer (MASCC) risk index has been utilized to determine the risk for poor clinical outcomes in patients with febrile neutropenia (FN) in an emergency center (EC). However, this index comprises subjective elements and elaborated metrics limiting its use in ECs. We sought to determine whether procalcitonin (PCT) level (biomarker of bacterial infection) with or without lactate level (marker of inadequate tissue perfusion) offers a potential alternative to MASSC score in predicting the outcomes of patients with FN presenting to an EC.

**Methods:**

We retrospectively identified 550 cancer patients with FN who presented to our EC between April 2018, and April 2019, and had serum PCT and lactate levels measured.

**Results:**

Compared with patients with PCT levels <0.25 ng/ml, those with levels ≥0.25 ng/ml had a significantly higher 14‐day mortality rate (5.2% vs. 0.7%; *p* = 0.002), a higher bloodstream infection (BSI) rate, and a longer hospital length of stay (LOS). Logistic regression analysis showed that patients with PCT levels ≥0.25 ng/ml and lactate levels >2.2 mmol/L were more likely to be admitted and have an LOS >7 days, BSI, and 14‐day mortality than patients with lower levels. PCT level was a significantly better predictor of BSI than MASSC score (*p* = 0.003) or lactate level (*p* < 0.0001).

**Conclusions:**

Procalcitonin level is superior to MASCC index in predicting BSI. The combination of PCT and lactate levels is a good predictor of BSI, hospital admission, and 14‐day mortality and could be useful in identifying high‐risk FN patients who require hospital admission.

## Graphical abstract

Procalcitonin can be used as a more objective and practical alternative to the Multinational Association for Supportive Care in Cancer score to predict the outcomes of cancer patients with febrile neutropenia in an emergency center.

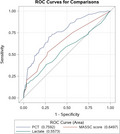

## 1 INTRODUCTION

Cancer patients receiving immunosuppressive therapy are prone to grave and potentially fatal complications, including infections and death. The proper evaluation, risk assessment, and management of these patients are crucial to avoid poor clinical outcomes, avert the patient's clinical deterioration, and prevent death.^1^ Physicians who first evaluate such patients must make critical decisions pertaining to treatment administration as well as ultimate patient disposition. These physicians must quickly determine whether their patients require intravenous or oral antibiotics or hospital admission. Identifying factors that can objectively predict patient outcomes may help physicians recognize high‐risk patients and optimize patient disposition. Hence, in a busy emergency center (EC), assessing the risk of cancer patients presenting with neutropenic fever can be challenging.

The Multinational Association for Supportive Care in Cancer (MASCC) risk index has been used to assess the risk of patients who present with chemotherapy‐induced febrile neutropenia (FN) to help determine patients’ management, need for hospitalization, and intravenous antibiotic therapy.^2^ An MASCC score <21 predicts a high risk for complications and indicates the need for admission. However, the MASCC risk index can be difficult to use in a busy EC. Furthermore, some elements of the index can be confusing (e.g., active chronic bronchitis) and others, such as the illness severity, are subject to physician interpretation.

Procalcitonin (PCT) level, a biomarker of bacterial infection and sepsis,^3^ has been used along with clinical judgment to guide antibiotic therapy in antibiotic stewardship programs, particularly for patients with lower respiratory tract infections.^4^, ^5^, ^6^ Serial measurements of PCT levels may have a prognostic role, as their failure to decrease may predict mortality.^7^ Although PCT levels may be elevated in cancer patients, they can further increase in febrile cancer patients in the setting of bacteremia or sepsis.^8^ PCT has been shown to improve the performance of the MASCC index in identifying patients with bacteremia or septic shock, particularly when used in low‐risk patients with FN.^9^

Lactate level has also been used as a prognostic biomarker of severe sepsis. A lactate level >4 mmol/L has been associated with increased mortality.^10^, ^11^ In hemodynamically stable patients, elevated lactate levels have preceded the progression to septic shock within 48 h. Like PCT levels, lactate levels are also elevated in cancer patients.^12^, ^13^, ^14^, ^15^ In patients with hematological malignancies hospitalized for FN, serum lactate levels ≥2 mmol/L have been associated with septic shock.^16^

The objective of this study was to determine whether serum PCT level alone or in combination with lactate level can serve as an alternative to MASCC risk index in predicting bloodstream infection (BSI), hospitalization, and 14‐day mortality in cancer patients with FN presenting to the EC.

## 2 METHODS

This retrospective study included all cancer patients who were evaluated in our EC for FN from 1 April 2018, to 30 April 2019, and whose serum PCT and lactate levels were measured at presentation.

MASCC scores were calculated using data obtained from patients' records. For burden of illness, all patients who presented to the EC were considered to have either moderate or severe symptoms. All patients were considered to be outpatients because they all presented to the EC at the time of neutropenic fever onset. All patients received parenteral fluids and were therefore considered to have dehydration.

Data were extracted from the institution's electronic medical records and included patients’ demographics (age, sex, race, and type of underlying cancer), laboratory test results (white blood cell count, absolute neutrophil count, PCT level, lactate level, and C‐reactive protein level), presence of BSI, and MASSC score. We also collected data on patients' hospitalization, inpatient length of stay (LOS), and 14‐ and 30‐day mortality.

Our Institutional Review Board approved the study protocol and a waiver of informed consent was obtained.

### 2.1 Definitions

Fever was defined as either a measured temperature of ≥100.4°F or a fever reported at home.

Neutropenia was defined as an absolute neutrophil count ≤500 cells/ml according to the Infectious Diseases Society of America 2011 clinical practice guideline for the use of antimicrobial agents in cancer patients with neutropenia.

Bloodstream infection was defined by the presence of a positive blood culture associated with fever.

### 2.2 Statistical analysis

We used the χ^2^ or Fisher exact test, as appropriate, to compare categorical variables. We used Wilcoxon rank‐sum tests to compare continuous variables because of the deviation of the data from the normal distribution. We assessed and compared the diagnostic performance of PCT levels, lactate levels, and MASCC scores for the prediction of the various outcomes. First, the area under the receiver operating characteristic (ROC) curve was evaluated and compared between the biomarkers. Then, using the optimal cut‐off value of each biomarker, the sensitivity, specificity, positive predictive values, and negative predictive values (NPVs) were calculated based on their definitions. For PCT level, we selected the previously suggested cut‐off value of 0.25 ng/ml, which has been used in different algorithms.^4^ Serum lactate levels >2.2 mmol/L were considered elevated.^15^ Last, multivariate logistic regression analysis was used to identify the independent predictors of hospital admission, LOS >7 days, BSI, and 14‐day mortality. The following factors were included in each analysis: age, sex, race, type of underlying malignancy, PCT level (<0.25 or ≥0.25 ng/ml), lactate level (≤2.2 or >2.2 mmol/L), and MASCC score (<21 or ≥21). All tests were two‐sided at a significance level of 0.05. The statistical analyses were performed using SAS version 9.3 (SAS Institute, Inc.).

## 3 RESULTS

We identified 550 cancer patients with FN who were evaluated in our EC and had serum PCT and lactate levels measured upon presentation. These patients' demographic and clinical characteristics are presented in Table 1. Most patients had hematological malignancies (70%) and were admitted to the hospital (80%) for a median LOS of 5 days. A BSI was identified in 116 patients (21%); BSI was due to gram‐negative organisms in 72 patients (66%) and gram‐positive organisms in 33 patients (30%).

**TABLE 1:** Patient characteristics

Characteristic	Patients (*N* = 550)
Age, median (IQR), years	56 (3–95)
Sex, male	285 (52)
Race	
White	389/541 (72)
African American	45/541 (8)
Asian	36/541 (7)
Other	71/541 (13)
Unknown	9
Type of cancer	
Hematological malignancy	386 (70)
Leukemia	214 (39)
Lymphoma	79 (14)
Multiple myeloma	4 (1)
Stem cell transplant	87 (16)
Other	2 (0.4)
Solid tumor	164 (30)
Brain and spine cancer	5 (1)
Breast cancer	27 (5)
Endocrine cancer	1 (0.2)
Gastrointestinal cancer	7 (1)
Gynecologic cancer	27 (5)
Head and neck cancer	10 (2)
Hepatobiliary cancer	3 (0.6)
Melanoma	5 (1)
Sarcoma	62 (11)
Thoracic cancer	17 (3)
Procalcitonin level, median (IQR), ng/ml	0.24 (0.13–0.64)
WBC count, median (IQR), cells/ml	400 (1001–900)
ANC, median (IQR), cells/ml	100 (30–220)
Lactate level, median (IQR), mmol/L	1.4 (0.95–1.8)
MASSC score, median (IQR)	19 (17–21)
CRP level, median (IQR), mg/L	101.2 (55.9–186.6)
BSI	116/544 (21)
Gram staining of organisms	
Gram‐positive	33/109 (30)
Gram‐negative	72/109 (66)
Both Gram‐positive and ‐negative	4/109 (4)
Hospital admission	440 (80)
Inpatient LOS, median (IQR), days	5 (4–9)
14‐day mortality	16/548 (3)
30‐day mortality	32/548 (6)

Note

Data are presented as no. patients (%) unless otherwise indicated.

Abbreviations: ANC, absolute neutrophil count; BSI, bloodstream infection; CRP, C‐reactive protein; EC, emergency center; IQR, interquartile range; LOS, length of stay; MASSC, Multinational Association for Supportive Care in Cancer; WBC, white blood cell.

The outcomes of patients according to their PCT levels, lactate levels, and MASCC scores are given in Table 2. In this cohort, 280 patients had PCT levels <0.25 ng/ml, and 270 had PCT levels ≥0.25 ng/ml; 452 patients had lactate levels ≤2.2 mmol/L, and 77 patients had lactate levels >2.2 mmol/L; 217 patients had MASCC scores ≥21 (low‐risk patients), and 333 patients had MASCC scores <21 (high‐risk patients). Patients with PCT levels ≥0.25 ng/ml were more likely to have a BSI than those with PCT levels <0.25 ng/ml (34% vs. 9%; *p* < 0.0001). Similarly, they were more likely to be hospitalized (85% vs. 75%; *p* = 0.006) and have an LOS >7 days (36% vs. 18%; *p* < 0.0001), have a higher 14‐day mortality rate (5.2% vs. 0.7%; *p* = 0.002), and have a higher 30‐day mortality rate (9.3% vs. 2.5%; *p* < 0.001). Patients with lactate levels >2.2 mmol/L were more likely to have a BSI than those with lactate levels <2.2 mmol/L (32% vs. 19%; *p* = 0.007). Similarly, they were more likely to have an LOS >7 days (42% vs. 24%; *p* = 0.002), a higher 14‐day mortality rate (13% vs. 1%; *p* < 0.0001), and higher 30‐day mortality rate (22% vs. 3%; *p* < 0.0001). Patients with MASCC scores <21 were more likely to have a BSI than those with MASCC scores ≥21 (26% vs. 14%; *p* < 0.001). Similarly, they were more likely to have an LOS >7 days (30% vs. 22%; *p* = 0.03), a higher 14‐day mortality rate (4.8% vs. 0%; *p* = 0.001), and a higher 30‐day mortality rate (9.4% vs. 0.5%; *p* < 0.0001).

**TABLE 2:** Patients’ outcomes according to procalcitonin (PCT) level, lactate level, and Multinational Association for Supportive Care in Cancer (MASCC) score

Outcome			*p* value
PCT level	<0.25 ng/ml (*n* = 280)	≥0.25 ng/ml (*n* = 270)	
BSI	26/277 (9)	90/267 (34)	<0.0001
Hospital admission	211 (75)	229 (85)	0.006
LOS >7 days	51 (18)	96 (36)	<0.0001
14‐day mortality	2 (0.7)	14/268 (5.2)	0.002
30‐day mortality	7 (2.5)	25/268 (9.3)	<0.001

Lactate level^a^	≤2.2 mmol/L (*n* = 452)	>2.2 mmol/L (*n* = 77)	
BSI	84/446 (19)	25 (32)	0.007
Hospital admission	355 (79)	67 (87)	0.09
LOS >7 days	110 (24)	32 (42)	0.002
14‐day mortality	5/450 (1)	10 (13)	<0.0001
30‐day mortality	14/450 (3)	17 (22)	<0.0001

MASSC score	<21 (*n* = 333)	≥21 (*n* = 217)	
BSI	86/327 (26)	30 (14)	<0.001
Hospital admission	275 (83)	165 (76)	0.06
LOS >7 days	100 (30)	47 (22)	0.03
14‐day mortality	16/331 (4.8)	0 (0)	0.001
30‐day mortality	31/331 (9.4)	1 (0.5)	<0.0001

Note

Data are presented as no. patients (%) unless otherwise indicated.

Abbreviation: BSI, bloodstream infection; LOS, length of stay.

^a^ Lactate level data were missing for 21 patients.

**FIGURE 1:**
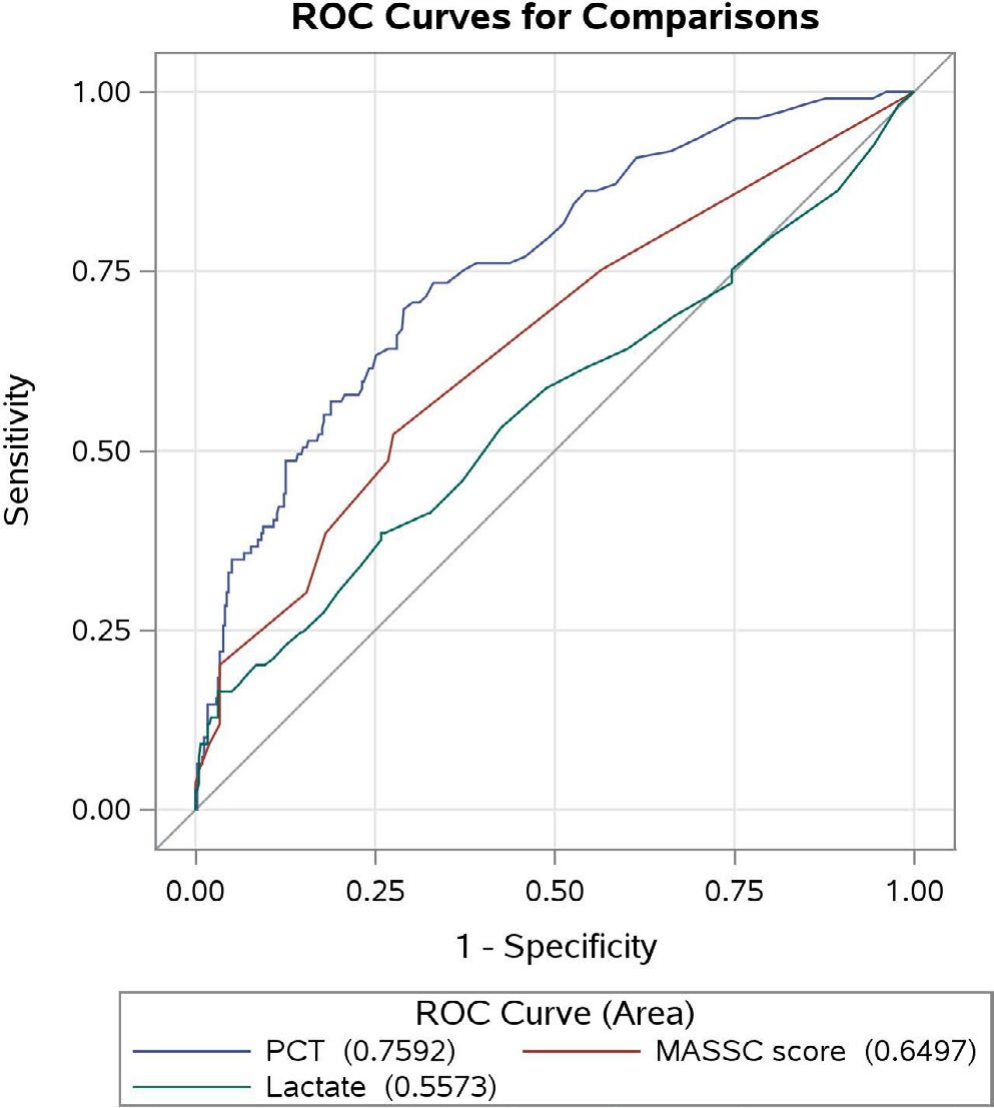
ROC curves for the prediction of BSI by PCT level, lactate level, and MASSC score. PCT level was a significantly better predictor of BSI than MASSC score (*p* = 0.003) or lactate level (*p* < 0.0001). For predicting BSI, the areas under the ROC curves were 0.76 (95% CI, 0.71–0.81) for PCT level, 0.65 (95% CI, 0.59–0.71) for MASSC score, and 0.56 (95% CI, 0.49–0.62) for lactate level. BSI, bloodstream infection; CI, confidence interval; MASSC, Multinational Association for Supportive Care in Cancer; PCT, procalcitonin; ROC, receiver‐operating characteristic

The results of the ROC analysis are shown in Figure 1. PCT level was a significantly better predictor of BSI than MASSC score (*p* = 0.003) or lactate level (*p* < 0.0001) (Figure 1). For predicting BSI, the areas under the ROC curves were 0.76 (95% confidence interval [CI], 0.71–0.81) for PCT level, 0.65 (95% CI, 0.59–0.71) for MASSC score, and 0.56 (95% CI, 0.49–0.62) for lactate level.

The diagnostic performances of PCT level, lactate level, and MASCC score for the prediction of patient outcomes are shown in Table 3. PCT level ≥0.25 ng/ml alone had a sensitivity of 0.78 and an NPV of 0.91 for BSI, whereas MASCC score had a sensitivity of 0.74 and an NPV of 0.86. In addition, the combination of PCT level ≥0.25 ng/ml plus lactate level >2.2 mmol/L had a sensitivity of 0.77 and an NPV of 0.90 for the prediction of BSI and a sensitivity of 0.93 and an NPV of 1.00 for the prediction of 14‐day mortality.

**TABLE 3:** Diagnostic performances of procalcitonin (PCT) level, lactate level, and Multinational Association for Supportive Care in Cancer (MASSC) score for predicting patients’ outcomes

Outcome	PCT level ≥0.25 ng/ml	Lactate level >2.2 mmol/L	PCT level ≥0.25 ng/ml and lactate level >2.2 mmol/L	MASSC score <21
BSI				
Sensitivity (95% CI)	0.78 (0.69–0.84)	0.23 (0.16–0.32)	0.77 (0.68–0.84)	0.74 (0.65–0.81)
Specificity (95% CI)	0.59 (0.54–0.63)	0.87 (0.84–0.90)	0.54 (0.50–0.59)	0.44 (0.39–0.48)
PPV (95% CI)	0.34 (0.28–0.40)	0.32 (0.23–0.44)	0.31 (0.26–0.36)	0.26 (0.22–0.31)
NPV (95% CI)	0.91 (0.87–0.94)	0.81 (0.77–0.85)	0.90 (0.86–0.93)	0.86 (0.81–0.90)
14‐day mortality				
Sensitivity (95% CI)	0.88 (0.64–0.97)	0.67 (0.42–0.85)	0.93 (0.70–0.99)	1.00 (0.81–1.00)
Specificity (95% CI)	0.52 (0.48–0.56)	0.87 (0.84–0.90)	0.49 (0.45–0.54)	0.41 (0.37–0.45)
PPV (95% CI)	0.05 (0.03–0.09)	0.13 (0.07–0.22)	0.05 (0.03–0.08)	0.05 (0.03–0.08)
NPV (95% CI)	0.99 (0.97–1.00)	0.99 (0.97–1.00)	1.00 (0.98–1.00)	1.00 (0.98–1.00)

Abbreviations: BSI, bloodstream infection; CI, confidence interval; NPV, negative predictive value; PPV, positive predictive value.

The results of the multivariate logistic regression analysis for predictors of outcomes are given in Table 4. PCT level ≥0.25 ng/ml was an independent predictor of hospital admission (odds ratio [OR], 1.62; 95% CI, 1.04–2.52), LOS >7 days (OR, 2.02; 95% CI, 1.33–3.08), and BSI (OR, 4.42; 95% CI, 2.73–7.18). Similarly, lactate level >2.2 mmol/L was an independent predictor of LOS >7 days (OR, 1.75; 95% CI, 1.02–3.01) and 14‐day mortality (OR, 10.78; 95% CI, 3.71–31.29), whereas MASCC score <21 was an independent predictor of only BSI (OR, 1.71; 95% CI, 1.06–2.78) and not hospital admission, LOS >7 days, or 14‐day mortality.

**TABLE 4:** Results of the multivariate logistic regression analysis for predictors of outcomes

Outcome	OR (95% CI)	*p* value
Hospital admission		
Type of cancer		<0.0001
Hemtological malignancy	3.34 (2.16–5.16)	
Solid tumor	Reference	
PCT level, ng/ml		0.033
<0.25	Reference	
≥0.25	1.62 (1.04–2.52)	
LOS >7 days		
Type of cancer		<0.0001
Hematological malignancy	5.49 (2.98–10.14)	
Solid tumor	Reference	
Lactate level, mmol/L		0.042
≤2.2	Reference	
>2.2	1.75 (1.02–3.01)	
PCT level, ng/ml		0.001
<0.25	Reference	
≥0.25	2.02 (1.33–3.08)	
BSI		
Type of cancer		0.002
Hematological malignancy	2.51 (1.41–4.45)	
Solid tumor	Reference	
PCT level, ng/ml		<0.0001
<0.25	Reference	
≥0.25	4.42 (2.73–7.18)	
MASSC score		0.03
<21	1.71 (1.06–2.78)	
≥21	Reference	
14‐day mortality		
Age, years		
≤55	Reference	
>55	8.17 (1.51–44.26)	0.015
Lactate level, mmol/L		<0.001
≤2.2	Reference	
>2.2	10.78 (3.71–31.29)	

Abbreviations: BSI, bloodstream infection; CI, confidence interval; EC, emergency center; LOS, length of stay; MASSC, Multinational Association for Supportive Care in Cancer; OR, odds ratio; PCT, procalcitonin.

## 4 DISCUSSION

We found that in cancer patients with FN, a PCT level ≥0.25 ng/ml is a good predictor of hospital admission, LOS >7 days, and BSI. In addition, a lactate level >2.2 ng/ml is a good predictor of LOS >7 days and 14‐day mortality. A MASCC score <21 was a predictor of only BSI, and a PCT level ≥0.25 ng/ml was a better predictor of BSI than the MASCC score. Hence, the combination of PCT and lactate levels is a good predictor of essential outcomes such as hospital admission, LOS, BSI, and 14‐day mortality and could therefore serve as a better and quicker alternative to the complicated and time‐consuming MASCC score in dictating the management and disposition of patients with FN presenting to the oncological EC.

Patients with hematological malignancies and patients who have received hematopoietic cell transplantation who present to the EC with FN are thoroughly evaluated, but their management can be challenging. Given the potential for serious complications, including death, it is critical to evaluate these patients—particularly high‐risk patients—and initiate appropriate empiric therapy quickly. Although an infectious source can be clinically identified or microbiologically documented in only 20%–30% of patients with FN,^17^ most patients with FN are admitted to the hospital to receive intravenous empirical antimicrobial therapy for a potentially life‐threatening event. Like previous reports, our study showed that 21% of patients with FN had a microbiologically documented BSI.^17^ However, 80% of all patients with FN (of whom 76% had an underlying hematological malignancy) were admitted to the hospital for intravenous antimicrobial therapy.

PCT level is a sensitive laboratory biomarker that increases in response to an infectious process within 3–6 h.^18^, ^19^, ^20^, ^21^ In patients with sepsis, PCT levels are correlated with disease severity.^21^, ^22^ PCT level, a measurement of which can be obtained within hours, has been used in treatment algorithms along with clinical judgment to guide antimicrobial therapy and reduce the prolonged and unnecessary use of antibiotics in patients with lower respiratory tract infections.^6^, ^23^, ^24^, ^25^, ^26^ Several PCT level cut‐off values have been evaluated; a PCT level of 0.25 ng/ml has often been used in different treatment algorithms. This cut‐off value was used in previous studies because patients who have acute respiratory infections and PCT levels <0.25 ng/ml are unlikely to have a bacterial infection.^4^ PCT level is also a good predictor of bacteremia. In a study of 925 patients with community‐acquired pneumonia, patients with PCT levels <0.25 ng/ml had a very low risk (<1%) for a positive blood culture.^27^ In addition, using PCT levels in treatment algorithms has been shown to reduce antimicrobial use and mortality in critically ill patients.^25^

The role of PCT level as a biomarker of bacterial infection has been extensively evaluated in the general population, and limited studies in immunocompromised cancer patients have shown promising results.^8^, ^28^ In addition to predicting cancer progression in non‐febrile cancer patients, PCT level can predict bacteremia and sepsis in febrile cancer patients.^8^ Monitoring PCT levels can also have a prognostic role in febrile cancer patients. Decreasing PCT levels have been associated with a successful response to antimicrobial therapy in cancer patients with and without neutropenia.^29^, ^30^ Furthermore, we previously showed that febrile cancer patients with decreasing PCT levels may not require a prolonged course of antimicrobial therapy.^28^ Therefore, PCT level kinetics in adjunction to clinical management could be used as an antimicrobial stewardship tool in febrile cancer patients. In addition to predicting response to antimicrobial therapy, PCT level has been shown to be a good predictor of BSI and mortality in critically ill febrile cancer patients.^31^

In our current study, <1% of patients with PCT <0.25 ng/ml died within 14 days compared to 5.2% in patients with PCT ≥0.25 ng/ml (*p* = 0.002). Similarly, only 1% of patients whose lactate levels were ≤2.2 mmol/L died within 14 days. A serum lactate level >4 mmol/l has been used as a risk factor for mortality in patients who present to an EC with suspected infection.^32^ In our study, multivariate analysis revealed that an elevated lactate level is associated with a 10‐fold increase in 14‐day mortality. Therefore, serum PCT levels and serum lactate levels seem to be good predictors of essential clinical outcomes such as BSI and 14‐day mortality. Furthermore, our multivariate analysis showed that a PCT level ≥0.25 ng/ml is a strong predictor of BSI, hospital admission, and LOS >7 days and a better predictor of BSI than MASCC score.

Although the MASCC risk index has been used to estimate the risk for complications in cancer patients with FN, it includes some elements that could be confusing (e.g., active chronic bronchitis) and other that are subjective (e.g., burden of illness).^2^ Moreover, calculating the MASCC score can be complicated, labor‐intensive, and time‐consuming, particularly in a busy EC. Furthermore, as shown in our study, MASCC score is less predictive of BSI, hospital admission, LOS, or death than the combination of PCT level plus lactate level is. In addition, MASCC score does not account for the duration and degree of neutropenia. All these factors make the MASCC score of limited use in a busy EC and make PCT level with or without lactate level a better alternative, particularly if these results are readily available.

Our study had several limitations. First, its observational retrospective design may have masked confounding variables. Documented infections, mainly BSIs, were based on positive cultures; however, cancer patients with neutropenia may have negative cultures or no obvious source of infection as a result of their immunosuppression. Therefore, documented infections may have been underestimated. Second, patients may have received a widespread use of different antibiotics and granulocyte colony‐stimulating factors that may have impacted their outcome. In addition, this was a single‐center study in a cancer center, which may limit the generalization of its results. Nevertheless, the major strength of this study was its large number of patients, most of whom had hematological malignancies, who were included for their neutropenic febrile episode.

In conclusion, in cancer patients with FN, a PCT level ≥0.25 ng/ml is a better predictor of BSI than MASSC score or lactate level. In addition, PCT is a good predictor of hospital admission and LOS >7 days. Whereas MASCC score is less predictive of BSI, a serum lactate level >2.2 mmol/L seems to be an excellent predictor of 14 day mortality. The combination of serum PCT level and serum lactate level may predict a wider spectrum of outcomes than the MASCC score index can. This combination could be useful in a busy EC to identify high‐risk febrile patients requiring hospital admission. This combination, which is based on the results of rapid and objective laboratory tests, is less labor‐intensive than the MASCC risk index, which is subject to assumptions and interpretations.

## CONFLICT OF INTEREST

We declare no competing interests.

## Data Availability

The study protocol, deidentified data, and generated tables and figures could be made available upon request by qualified researchers for well‐specified research purposes. Requests should be sent to achaftari@mdanderson.org and yijiang@mdanderson.org. Data will be made available upon request during the 6 months following the publication.
